# 2-Benz­yloxy-1-naphthaldehyde

**DOI:** 10.1107/S1600536809004486

**Published:** 2009-02-18

**Authors:** Rong Gao, Wen-Hong Li, Peng Liu, Ping-An Wang

**Affiliations:** a Department of Chemical Engineering, Northwest University, Taibai North Road 229, 710069 Xi-An, People’s Republic of China; b Department of Chemistry, School of Pharmacy, Fourth Military Medical University, Changle West Road 17, 710032 Xi-An, People’s Republic of China

## Abstract

In the title compound, C_18_H_14_O_2_, the dihedral angle between the phenyl and naphthyl ring systems is 21.8 (3)°. The packing of mol­ecules in the crystal structure is stabilized by weak inter­molecular C—H⋯O hydrogen bonds.

## Related literature

For the preparation of 2-benz­yloxy-1-naphthaldehyde, see: Quideau *et al.* (2001 ▶). For synthetic use of the title compound, see: Knight & Little (2001 ▶).
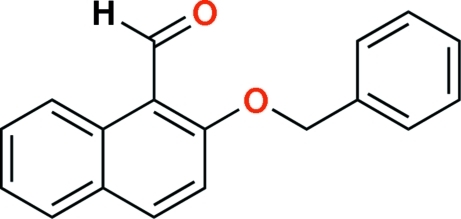


## Experimental

### 

#### Crystal data

C_18_H_14_O_2_
*M* *_r_* = 262.29
Monoclinic, 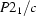

*a* = 10.427 (7) Å
*b* = 8.128 (6) Å
*c* = 15.787 (11) Å
β = 94.746 (11)°
*V* = 1333.3 (16) Å^3^
*Z* = 4
Mo *K*α radiation
μ = 0.08 mm^−1^
*T* = 296 K
0.39 × 0.26 × 0.16 mm

#### Data collection

Bruker SMART APEX CCD area-detector diffractometer
Absorption correction: multi-scan (**SADABS**; Bruker, 2005 ▶) *T*_min_ = 0.968, *T*_max_ = 0.987
5088 measured reflections
2262 independent reflections
1354 reflections with *I* > 2σ(*I*)
*R*_int_ = 0.038

#### Refinement

*R*[*F* ^2^ > 2σ(*F* ^2^)] = 0.084
*wR*(*F* ^2^) = 0.285
*S* = 1.04
2262 reflections
181 parameters
H-atom parameters constrained
Δρ_max_ = 0.41 e Å^−3^
Δρ_min_ = −0.53 e Å^−3^

### 

Data collection: *SMART* (Bruker, 2002 ▶); cell refinement: *SAINT* (Bruker, 2002 ▶); data reduction: *SAINT*; program(s) used to solve structure: *SHELXS97* (Sheldrick, 2008 ▶); program(s) used to refine structure: *SHELXL97* (Sheldrick, 2008 ▶); molecular graphics: *SHELXTL* (Sheldrick, 2008 ▶); software used to prepare material for publication: *Mercury* (Macrae *et al.*, 2006 ▶) and *CAMERON* (Watkin *et al.*, 1996 ▶).

## Supplementary Material

Crystal structure: contains datablocks I, global. DOI: 10.1107/S1600536809004486/wn2303sup1.cif

Structure factors: contains datablocks I. DOI: 10.1107/S1600536809004486/wn2303Isup2.hkl

Additional supplementary materials:  crystallographic information; 3D view; checkCIF report

## Figures and Tables

**Table 1:** Hydrogen-bond geometry (Å, °)

*D*—H⋯*A*	*D*—H	H⋯*A*	*D*⋯*A*	*D*—H⋯*A*
C12—H12*A*⋯O1^i^	0.97	2.48	3.381 (4)	155
C14—H14⋯O1^i^	0.93	2.72	3.544 (5)	148

Symmetry code: (i) 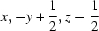.

**Fig. 1.**
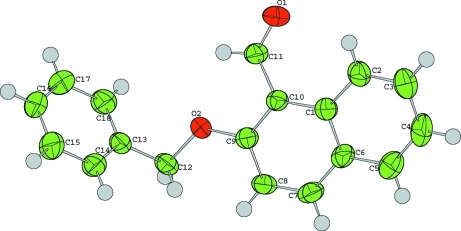
The molecular structure of the title compound, showing the atom-labelling scheme. Displacement ellipsoids are drawn at the 50% probability level. Hydrogen atoms are drawn as spheres of arbitrary radius.

**Fig. 2.**
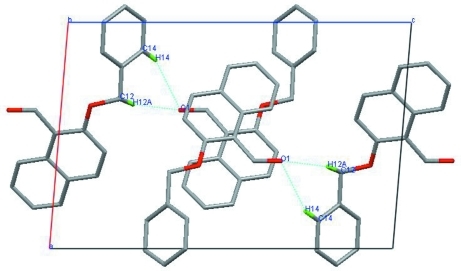
The packing of the title compound, viewed down the b axis. Dotted lines indicate hydrogen bonds.

## supplementary crystallographic information

### Comment

The title compound, 2-benzyloxy-1-naphthaldehyde, was obtained by benzylation of 2-hydroxy-1-naphthaldehyde with benzyl bromide (Quideau *et al.*, 2001) and used for alkylation of position 4 in the naphthyl ring system. It has also been used for the intramolecular trapping of benzynes to yield some novel xanthenes (Knight & Little, 2001).

In the title compound, C_18_H_14_O_2_, the dihedral angle between the phenyl and naphthyl ring systems is  21.8 (3)°. The packing of molecules in the crystal structure is stabilized by weak intermolecular C—H···O hydrogen bonds.

### Experimental

To a stirred solution of commercially available 2-hydroxy-1-naphthaldehyde (4.30 g, 25.0 mmol) in *N*,*N*-dimethylformamide (100.0 cm^3^) was added potassium carbonate (3.82 g, 27.6 mmol) and benzyl bromide (3.0 cm^3^, 25.0 mmol), and the mixture was heated for 4 h at 90–100°C. The solution was filtered through celite and the solvent removed *in vacuo*. The residue was dissolved with Et_2_O (160 cm^3^), washed with 1 *M* NaOH (110 cm^3^), brine (2× 110 cm^3^), and dried over Na_2_SO_4_. Evaporation of the solvent afforded the title compound as a light yellow powder (6.0 g, 91%). The melting point and the spectroscopic data of the title compound were consisted with the reported literature (Quideau *et al.*, 2001).

### Refinement

All H atoms were placed in calculated positions and refined as riding, with C—H = 0.93–0.97Å and with *U*_iso_(H) = 1.2*U*_eq_(C). The values of R[F^2^>2σ(F^2^)] and wR(F^2^)  are 0.084 and 0.285, respectively; these high values  may be due to the poor quality of the crystals.

### Crystal data

**Table:**

C_18_H_14_O_2_	*F*(000) = 552
*M**_r_* = 262.29	*D*_x_ = 1.307 Mg m^−^^3^
Monoclinic, *P*2_1_/*c*	Melting point: 393(1) K
Hall symbol: -P 2ybc	Mo *K*α radiation, λ = 0.71073 Å
*a* = 10.427 (7) Å	Cell parameters from 1554 reflections
*b* = 8.128 (6) Å	θ = 2.6–24.3°
*c* = 15.787 (11) Å	µ = 0.08 mm^−^^1^
β = 94.746 (11)°	*T* = 296 K
*V* = 1333.3 (16) Å^3^	Block, colourless
*Z* = 4	0.39 × 0.26 × 0.16 mm

### Data collection

**Table:**

Bruker SMART APEX CCD area-detector diffractometer	2262 independent reflections
Radiation source: fine-focus sealed tube	1354 reflections with *I* > 2σ(*I*)
graphite	*R*_int_ = 0.038
φ and ω scans	θ_max_ = 25.1°, θ_min_ = 2.0°
Absorption correction: multi-scan (*SADABS*; Bruker, 2005)	*h* = −7→12
*T*_min_ = 0.968, *T*_max_ = 0.987	*k* = −9→6
5088 measured reflections	*l* = −18→17

### Refinement

**Table:**

Refinement on *F*^2^	Primary atom site location: structure-invariant direct methods
Least-squares matrix: full	Secondary atom site location: difference Fourier map
*R*[*F*^2^ > 2σ(*F*^2^)] = 0.084	Hydrogen site location: inferred from neighbouring sites
*wR*(*F*^2^) = 0.285	H-atom parameters constrained
*S* = 1.04	*w* = 1/[σ^2^(*F*_o_^2^) + (0.18*P*)^2^ + 0.612*P*] where *P* = (*F*_o_^2^ + 2*F*_c_^2^)/3
2262 reflections	(Δ/σ)_max_ < 0.001
181 parameters	Δρ_max_ = 0.41 e Å^−^^3^
0 restraints	Δρ_min_ = −0.53 e Å^−^^3^

### Special details

**Table:**

Geometry. All e.s.d.'s (except the e.s.d. in the dihedral angle between two l.s. planes) are estimated using the full covariance matrix. The cell e.s.d.'s are taken into account individually in the estimation of e.s.d.'s in distances, angles and torsion angles; correlations between e.s.d.'s in cell parameters are only used when they are defined by crystal symmetry. An approximate (isotropic) treatment of cell e.s.d.'s is used for estimating e.s.d.'s involving l.s. planes.
Refinement. Refinement of *F*^2^ against ALL reflections. The weighted *R*-factor *wR* and goodness of fit *S* are based on *F*^2^, conventional *R*-factors *R* are based on *F*, with *F* set to zero for negative *F*^2^. The threshold expression of *F*^2^ > σ(*F*^2^) is used only for calculating *R*-factors(gt) *etc*. and is not relevant to the choice of reflections for refinement. *R*-factors based on *F*^2^ are statistically about twice as large as those based on *F*, and *R*- factors based on ALL data will be even larger.

### Fractional atomic coordinates and isotropic or equivalent isotropic displacement parameters (Å^2^)

**Table:**

	*x*	*y*	*z*	*U*_iso_*/*U*_eq_	
O1	0.6120 (3)	0.2057 (4)	0.65206 (15)	0.0658 (10)	
O2	0.6321 (2)	0.1063 (4)	0.41483 (14)	0.0531 (8)	
C1	0.4002 (3)	0.3376 (5)	0.5290 (2)	0.0411 (9)	
C2	0.3808 (4)	0.4033 (5)	0.6101 (2)	0.0518 (11)	
H2	0.4423	0.3837	0.6551	0.062*	
C3	0.2749 (4)	0.4942 (6)	0.6241 (3)	0.0630 (12)	
H3	0.2641	0.5335	0.6784	0.076*	
C4	0.1821 (4)	0.5289 (7)	0.5572 (3)	0.0716 (14)	
H4	0.1103	0.5918	0.5671	0.086*	
C5	0.1967 (4)	0.4710 (5)	0.4783 (3)	0.0577 (11)	
H5	0.1349	0.4952	0.4343	0.069*	
C6	0.3053 (3)	0.3737 (6)	0.4616 (2)	0.0504 (11)	
C7	0.3228 (3)	0.3153 (5)	0.3794 (2)	0.0523 (11)	
H7	0.2608	0.3395	0.3354	0.063*	
C8	0.4275 (3)	0.2244 (6)	0.3622 (2)	0.0539 (11)	
H8	0.4354	0.1851	0.3075	0.065*	
C9	0.5238 (3)	0.1904 (5)	0.4280 (2)	0.0431 (10)	
C10	0.5100 (3)	0.2424 (5)	0.5110 (2)	0.0392 (9)	
C11	0.6122 (3)	0.1927 (6)	0.5756 (2)	0.0514 (11)	
H11	0.6853	0.1459	0.5556	0.062*	
C12	0.6621 (3)	0.0652 (6)	0.3305 (2)	0.0575 (12)	
H12A	0.6381	0.1548	0.2918	0.069*	
H12B	0.6154	−0.0326	0.3108	0.069*	
C13	0.8049 (3)	0.0350 (5)	0.3337 (2)	0.0468 (10)	
C14	0.8757 (4)	0.1189 (6)	0.2765 (2)	0.0571 (12)	
H14	0.8348	0.1902	0.2368	0.069*	
C15	1.0079 (4)	0.0954 (7)	0.2792 (3)	0.0713 (15)	
H15	1.0549	0.1484	0.2397	0.086*	
C16	1.0699 (4)	−0.0047 (6)	0.3390 (3)	0.0635 (13)	
H16	1.1589	−0.0163	0.3412	0.076*	
C17	1.0006 (4)	−0.0884 (6)	0.3960 (3)	0.0630 (12)	
H17	1.0423	−0.1582	0.4360	0.076*	
C18	0.8681 (4)	−0.0675 (6)	0.3932 (2)	0.0579 (12)	
H18	0.8214	−0.1232	0.4319	0.069*	

### Atomic displacement parameters (Å^2^)

**Table:**

	*U*^11^	*U*^22^	*U*^33^	*U*^12^	*U*^13^	*U*^23^
O1	0.0596 (17)	0.099 (3)	0.0374 (14)	0.0078 (16)	−0.0065 (11)	−0.0031 (14)
O2	0.0498 (15)	0.075 (2)	0.0352 (13)	0.0178 (14)	0.0062 (10)	−0.0012 (12)
C1	0.0352 (18)	0.047 (3)	0.0415 (17)	−0.0085 (16)	0.0054 (14)	0.0049 (17)
C2	0.045 (2)	0.062 (3)	0.050 (2)	−0.003 (2)	0.0112 (16)	−0.0008 (19)
C3	0.058 (2)	0.066 (3)	0.068 (3)	−0.003 (2)	0.023 (2)	−0.001 (2)
C4	0.046 (2)	0.082 (4)	0.090 (3)	0.008 (2)	0.024 (2)	0.003 (3)
C5	0.043 (2)	0.056 (3)	0.075 (3)	−0.0013 (19)	0.0041 (18)	0.008 (2)
C6	0.0307 (17)	0.068 (3)	0.052 (2)	−0.0035 (17)	0.0040 (15)	0.011 (2)
C7	0.040 (2)	0.067 (3)	0.047 (2)	−0.0013 (19)	−0.0074 (15)	0.010 (2)
C8	0.045 (2)	0.082 (3)	0.0340 (18)	−0.002 (2)	−0.0007 (15)	0.0032 (18)
C9	0.0345 (17)	0.055 (3)	0.0393 (18)	−0.0040 (17)	0.0035 (13)	0.0078 (17)
C10	0.0328 (17)	0.047 (2)	0.0373 (17)	−0.0047 (15)	0.0012 (13)	0.0028 (16)
C11	0.0386 (19)	0.076 (3)	0.0386 (19)	0.0002 (19)	−0.0005 (14)	0.0038 (19)
C12	0.043 (2)	0.096 (4)	0.0337 (17)	−0.003 (2)	0.0069 (14)	−0.004 (2)
C13	0.0395 (18)	0.066 (3)	0.0355 (16)	−0.0014 (18)	0.0055 (14)	−0.0021 (18)
C14	0.045 (2)	0.081 (4)	0.047 (2)	0.005 (2)	0.0070 (16)	0.015 (2)
C15	0.044 (2)	0.107 (4)	0.064 (3)	0.003 (2)	0.0155 (19)	0.017 (3)
C16	0.043 (2)	0.087 (4)	0.061 (2)	0.010 (2)	0.0049 (18)	0.002 (2)
C17	0.056 (2)	0.075 (3)	0.057 (2)	0.015 (2)	−0.0031 (18)	0.008 (2)
C18	0.054 (2)	0.071 (3)	0.050 (2)	−0.002 (2)	0.0091 (17)	0.010 (2)

### Geometric parameters (Å, °)

**Table:**

O1—C11	1.211 (4)	C8—H8	0.9300
O2—C9	1.351 (4)	C9—C10	1.395 (5)
O2—C12	1.433 (4)	C10—C11	1.471 (5)
C1—C2	1.417 (5)	C11—H11	0.9300
C1—C6	1.422 (5)	C12—C13	1.506 (5)
C1—C10	1.430 (5)	C12—H12A	0.9700
C2—C3	1.362 (6)	C12—H12B	0.9700
C2—H2	0.9300	C13—C18	1.382 (6)
C3—C4	1.400 (6)	C13—C14	1.391 (5)
C3—H3	0.9300	C14—C15	1.389 (5)
C4—C5	1.351 (6)	C14—H14	0.9300
C4—H4	0.9300	C15—C16	1.367 (6)
C5—C6	1.424 (5)	C15—H15	0.9300
C5—H5	0.9300	C16—C17	1.380 (6)
C6—C7	1.407 (5)	C16—H16	0.9300
C7—C8	1.365 (5)	C17—C18	1.389 (5)
C7—H7	0.9300	C17—H17	0.9300
C8—C9	1.412 (5)	C18—H18	0.9300
			
C9—O2—C12	120.7 (3)	C9—C10—C11	116.3 (3)
C2—C1—C6	117.1 (3)	C1—C10—C11	123.8 (3)
C2—C1—C10	124.1 (3)	O1—C11—C10	127.3 (4)
C6—C1—C10	118.8 (3)	O1—C11—H11	116.3
C3—C2—C1	121.9 (4)	C10—C11—H11	116.3
C3—C2—H2	119.0	O2—C12—C13	107.3 (3)
C1—C2—H2	119.0	O2—C12—H12A	110.2
C2—C3—C4	120.4 (4)	C13—C12—H12A	110.2
C2—C3—H3	119.8	O2—C12—H12B	110.2
C4—C3—H3	119.8	C13—C12—H12B	110.2
C5—C4—C3	120.1 (4)	H12A—C12—H12B	108.5
C5—C4—H4	120.0	C18—C13—C14	119.2 (3)
C3—C4—H4	120.0	C18—C13—C12	122.4 (3)
C4—C5—C6	121.1 (4)	C14—C13—C12	118.3 (3)
C4—C5—H5	119.5	C15—C14—C13	119.4 (4)
C6—C5—H5	119.5	C15—C14—H14	120.3
C7—C6—C1	119.0 (3)	C13—C14—H14	120.3
C7—C6—C5	121.6 (3)	C16—C15—C14	121.0 (4)
C1—C6—C5	119.4 (4)	C16—C15—H15	119.5
C8—C7—C6	122.2 (3)	C14—C15—H15	119.5
C8—C7—H7	118.9	C15—C16—C17	120.0 (4)
C6—C7—H7	118.9	C15—C16—H16	120.0
C7—C8—C9	119.4 (3)	C17—C16—H16	120.0
C7—C8—H8	120.3	C16—C17—C18	119.5 (4)
C9—C8—H8	120.3	C16—C17—H17	120.2
O2—C9—C10	116.8 (3)	C18—C17—H17	120.2
O2—C9—C8	122.5 (3)	C13—C18—C17	120.8 (4)
C10—C9—C8	120.6 (3)	C13—C18—H18	119.6
C9—C10—C1	119.9 (3)	C17—C18—H18	119.6
			
C6—C1—C2—C3	1.4 (6)	O2—C9—C10—C11	−2.9 (5)
C10—C1—C2—C3	179.6 (4)	C8—C9—C10—C11	176.6 (4)
C1—C2—C3—C4	−1.4 (7)	C2—C1—C10—C9	−176.6 (4)
C2—C3—C4—C5	0.5 (7)	C6—C1—C10—C9	1.5 (5)
C3—C4—C5—C6	0.4 (7)	C2—C1—C10—C11	4.0 (6)
C2—C1—C6—C7	178.0 (4)	C6—C1—C10—C11	−177.9 (3)
C10—C1—C6—C7	−0.2 (6)	C9—C10—C11—O1	−169.9 (4)
C2—C1—C6—C5	−0.5 (5)	C1—C10—C11—O1	9.6 (7)
C10—C1—C6—C5	−178.8 (3)	C9—O2—C12—C13	158.2 (3)
C4—C5—C6—C7	−178.8 (4)	O2—C12—C13—C18	49.5 (6)
C4—C5—C6—C1	−0.3 (6)	O2—C12—C13—C14	−127.9 (4)
C1—C6—C7—C8	0.3 (6)	C18—C13—C14—C15	1.4 (6)
C5—C6—C7—C8	178.8 (4)	C12—C13—C14—C15	178.8 (4)
C6—C7—C8—C9	−1.6 (6)	C13—C14—C15—C16	−2.2 (7)
C12—O2—C9—C10	−172.4 (3)	C14—C15—C16—C17	2.2 (8)
C12—O2—C9—C8	8.1 (6)	C15—C16—C17—C18	−1.3 (7)
C7—C8—C9—O2	−177.6 (4)	C14—C13—C18—C17	−0.6 (7)
C7—C8—C9—C10	2.9 (6)	C12—C13—C18—C17	−177.9 (4)
O2—C9—C10—C1	177.6 (3)	C16—C17—C18—C13	0.5 (7)
C8—C9—C10—C1	−2.8 (6)		

### Hydrogen-bond geometry (Å, °)

**Table:**

*D*—H···*A*	*D*—H	H···*A*	*D*···*A*	*D*—H···*A*
C12—H12A···O1^i^	0.97	2.48	3.381 (4)	155
C14—H14···O1^i^	0.93	2.72	3.544 (5)	148

Symmetry codes: (i) *x*, −*y*+1/2, *z*−1/2.
